# Anaplastic lymphoma kinase rearrangement prevalence in patients with advanced non-small cell lung cancer in the United States: retrospective real world data

**DOI:** 10.18632/oncotarget.28114

**Published:** 2021-11-09

**Authors:** Timothy Craig Allen, Yan Xiao, Baiyu Yang, Denise Croix, Anup Abraham, Stella Redpath, Julia Engstrom-Melynk, Roma Shah, Jaya Madala, Eric H. Bernicker

**Affiliations:** ^1^Department of Pathology, University of Mississippi Medical Center, Jackson, MS, USA; ^2^Data Services, Roche Information Solutions, Pleasanton, CA, USA; ^3^Medical and Scientific Affairs, Roche Diagnostics Corporation, Indianapolis, IN, USA; ^4^Evidence Strategy, Genesis Research, Hoboken, NJ, USA; ^5^Cancer Center, Houston Methodist Hospital, Houston, TX, USA; ^6^Current affiliation: Digital Health, AstraZeneca R&D, Beijing, China; ^7^Current affiliation: Medical Diagnostics, AstraZeneca, Gaithersburg, MD, USA

**Keywords:** ALK rearrangement, NSCLC, prevalence

## Abstract

Objective: This study assessed the prevalence of anaplastic lymphoma kinase (ALK) rearrangements in US oncology practices.

Materials and Methods: Using a nationwide real-world database, we included adults with advanced non-small cell lung cancer (aNSCLC, stage IIIB- IV) diagnosed January 2015 – May 2019, with documented ALK testing results and smoking status. Rearrangement prevalence was assessed overall and then stratified by patient characteristics.

Results: The cohort included 19,895 eligible patients with a mean age 68.5 years, majority ever-smokers (85.5%) and from community centers (92.2%). The overall ALK rearrangement prevalence was 2.6%. Positivity rate varied by histology and smoking status; it was the highest among non-smoking patients with non-squamous histology (9.3%). Differences in ALK status also varied by age and race, with young patients (18–39 years) having a higher prevalence (21.6%) vs. older patients (age ≥55 = 2.2%); Asian patients had a prevalence of 6.3%. Patients that were positive for other mutations or rearrangements had a lower ALK positivity rate (0.5%) and patients positive for PD-L1 had a rate of 3.0%.

Conclusions: The likelihood of finding an ALK translocation was highest in younger patients and nonsmokers; however, age and smoking history were not discriminative enough to exclude testing based on clinical variables.

## INTRODUCTION

The ability to detect actionable alterations in patients with advanced lung cancer has revolutionized the field of thoracic oncology and is in part responsible for a significant decline in mortality [1]. However, uptake of routine testing as per NCCN guidelines has been slow for a variety of reasons, including small biopsies, tissue exhaustion from sequential testing and slow diffusion of knowledge [2]. Timely identification of patients who might benefit from tyrosine kinase inhibitors (TKIs) is crucial, as it is known that TKIs selected on the basis of a driver alteration gives patients a much better chance at prolonged response rather than getting treatment following systemic chemotherapy. In addition, it is now being recognized that immunotherapy drugs given sequentially before TKIs but not after are associated with a much higher rate of pneumonitis and other adverse events [3, 4].

ALK translocations are very sensitive to a number of well tolerated oral medications [5, 6]. Responding patients often have prolonged non-trivial survival benefit—a recent study found a median OS of 48 months in a cohort of patients treated mostly with an older generation of ALK TKIs [7]. Clinical predictors, while useful, have been recognized for a long time as being insufficient for deciding which patients with advanced pulmonary adenocarcinoma should be tested [8]. We performed a retrospective study of a database to acquire real-world clinical data on the frequency of the translocation in a large pool of patients drawn primarily from community hospitals and practices.

## RESULTS

### Patient characteristics

In total, 19,895 patients with aNSCLC diagnosed from 2015 to 2019 were included in this study. The mean age of patients was 68.5 ± 10.0 years. Men comprised 50.4% (10,029) of the patient cohort and 68.4% (13,599 of 19,895) of patients were Caucasian. A large proportion of patients had a non-squamous histology type (16,025 of 19,895 or 80.5%) and smoking history (17,003 of 19,895 or 85.5%), and the majority of patients were from community practices (18,350 of 19,895 or 92.2%) (Table 1).

**Table 1:** Prevalence of ALK rearrangement in aNSCLC according to patient characteristics

Characteristics	*N*	% Total patients	*N* ALK+ patients	% ALK+ by characteristic
**Total**	**19,985**	**100.0**	**519**	**2.6**
**Age at aNSCLC diagnosis^1^**				
18–39	125	0.6	27	21.6
40–54	1,627	8.2	85	5.2
55+	18,143	91.2	407	2.2
**Gender**				
Female	9,866	49.6	275	2.8
Male	10,029	50.4	244	2.4
**Race^1^**				
White	13,599	68.4	324	2.4
Black or African American	1,673	8.4	37	2.2
Asian	623	3.1	39	6.3
Others	2,043	10.3	43	2.1
Unknown	1,957	9.8	50	2.6
**Geographic Location^1^**				
Northeast	3,892	19.6	98	2.5
South	7,988	40.2	154	1.9
Midwest	2,973	14.9	83	2.8
West	3,143	15.8	108	3.4
Unknown	1,899	9.5	76	4.0
**Practice Type^1^**				
Community	18,350	92.2	452	2.5
Academic	1,545	7.8	67	4.3
**Smoking Status^1^**				
Nonsmoker	2,862	14.5	257	8.9
Smoker	17,003	85.5	262	1.5
**Histology^1^**				
Non-squamous	16,025	80.5	477	3.0
NSCLC NOS	903	4.5	17	1.9
Squamous	2,967	14.9	25	0.8
**PD-L1 Status**				
PD-L1 positive	1,839	9.2	56	3.0
PD-L1 negative/unknown	18,056	90.8	463	2.6
**Other Biomarker Status^1,2^**				
Any other biomarker positive	6,240	31.4	33	0.5
Other biomarker negative/unknown	13,655	6806	486	3.6

^1^Prevalence of ALK rearrangement was significantly different (*p* < 0.001). ^2^Other biomarkers include EGFR, ROS1, KRAS, BRAF.

### Prevalence of ALK rearrangement

**Figure 1:**
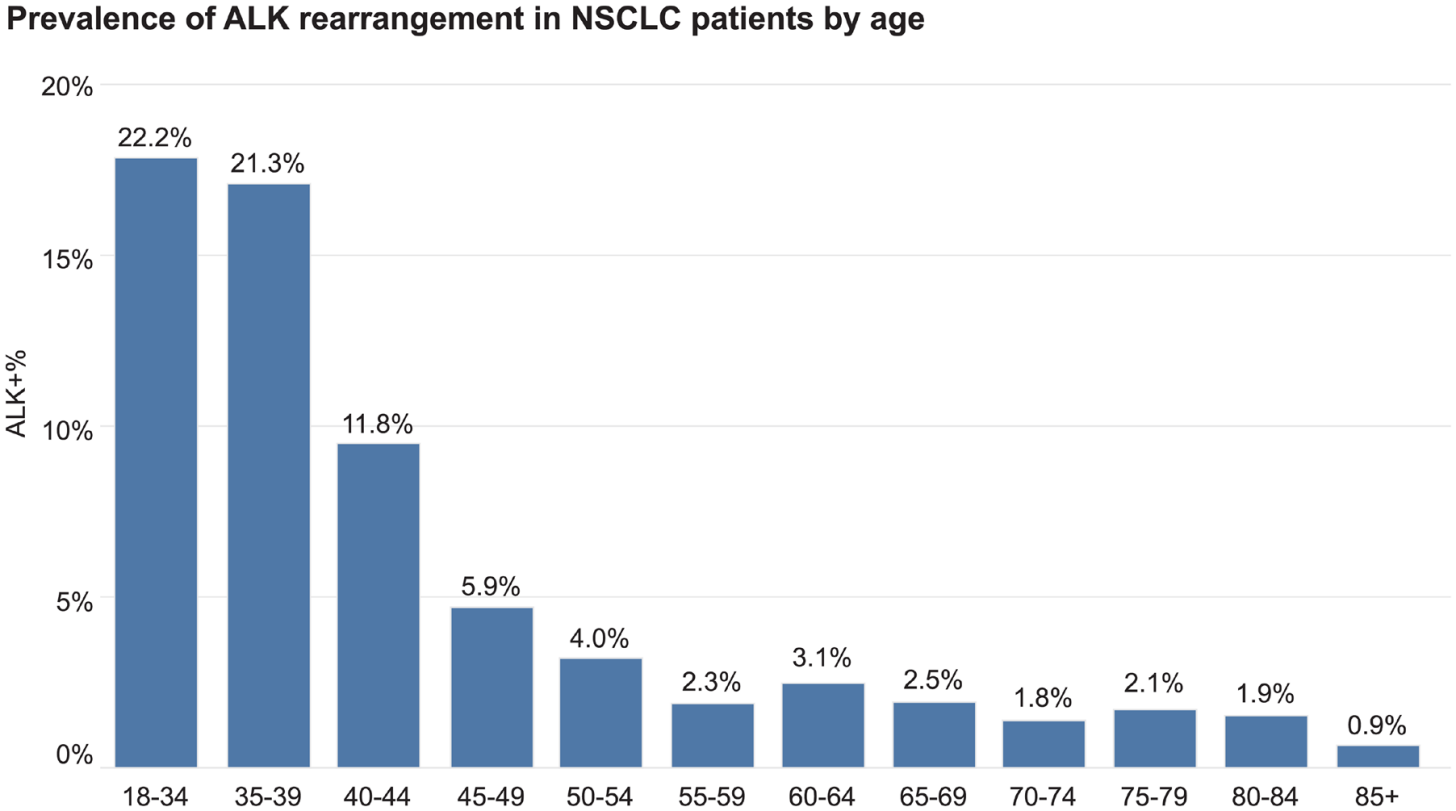
Prevalence of ALK rearrangement in aNSCLC patients by age.

Overall, the prevalence of ALK rearrangement was 2.6% in aNSCLC patients (519 of 19,895 patients). Prevalence varied by patients’ demographic characteristics. The rate of ALK rearrangement was the highest (>20%) among patients younger than 40 years old. It decreased to over 10% in patients in their early 40s, and further decreased to a plateau of approximately 2% in patients older than 55 years old (Figure 1). Asian patients had a higher ALK rearrangement rate (39 of 623 or 6.3%) than other patients. There were no significant differences in the number of ALK rearrangements found in females (275 of 9866 or 2.8%) compared to male patients (244 of 10,029 or 2.4%). The prevalence of ALK translocation was higher in patients from academic centers (4.3%) than patients from community centers (2.5%).

**Figure 2:**
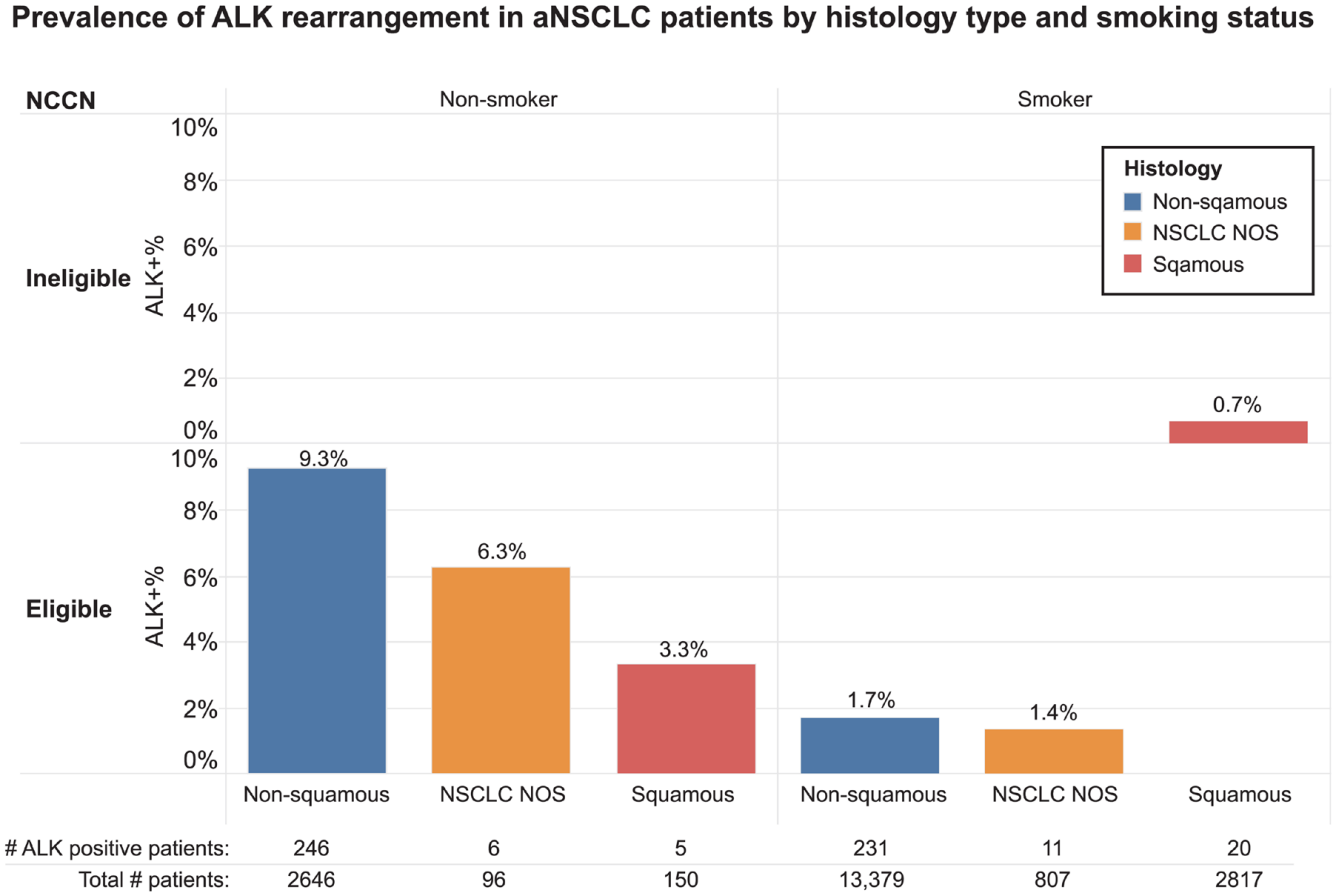
Prevalence of ALK rearrangement in aNSCLC patients by histology type and smoking status.

ALK rearrangement rates also varied by some patients’ clinical characteristics. Patients with no smoking history had a higher ALK rearrangement rate compared to those with a history of smoking (8.9% vs. 1.5%). Overall, patients with squamous histology type had a lower ALK rearrangement rate (0.8%) than patients with non-squamous (3.0%) and nonspecific (1.9%) histology types. However, there was a subgroup of patients with squamous histology and no history of smoking that had a relatively high ALK rearrangement rate (3.3%) (Figure 2). Results from this study showed that the ALK rearrangement rate in NCCN guideline ALK testing ineligible patients (those with squamous histology and a history of smoking) was 0.7% and ranged from 1.4% to 9.3% in eligible patients (Figure 2).

Patients who were positive for any other driver mutations or rearrangements (EGFR, KRAS, BRAF or ROS1) had a significantly lower ALK positivity rate (0.5%), while patients reported to be positive for PD-L1 had an ALK positivity rate of 3.0%.

## DISCUSSION

Since the first descriptions of ALK rearrangements in lung cancer, prevalence estimates for this rearrangement have ranged relatively broadly from <2–13% [9–12]. The overall ALK rearrangement rate (2.6%) in this large retrospective data set was within the range observed previously in other non-selected populations [13–15]. Likewise, the younger age of the patients whose cancers harbored the ALK translocation has been reported by other groups as well [16, 17]. It is not surprising that, of those young patients with advanced lung cancer, there would be an enriched population of ALK positive patients and that was confirmed in the evaluation of this dataset. One in five patients in the 18–34 year old age group were found to be ALK fusion positive (Figure 1 for reference). This was cut in half by ages 40–44 (11.8%). But even in patients over 55, there was a 2% rate of positivity where treatment would be substantially different if an ALK rearrangement was detected. The fact that the prevalence of ALK rearrangement was higher in patients from academic centers than patients from community centers (4.3% vs. 2.5%) could be explained by patient self-referral patterns. Also, since the majority of the data is from patients in community settings, this finding could be attributed to the limited number of patients in this database that were seen in academic centers.

While our understanding of molecular pathophysiology has developed, clinical features such as histology, age and smoking history have been utilized when determining testing for driver alterations. As such, we examined histology and smoking status within this patient cohort. Regardless of documented histology, a higher ALK rearrangement rate (8.9%) was observed among patients who had no smoking history compared to patients with a smoking history (1.5% ALK positivity) which represent the largest number of patients in this cohort (17,003). Analyzing patients in this study by their histology, we observed that patients with non-squamous histology had an ALK rearrangement rate of 3%, patients in the NSCLC NOS category (most likely poorly differentiated adenocarcinomas and squamous cell carcinomas) 1.9%, and patients with squamous histology 0.8%. It is not discernable in our data if the patients with squamous histology had small biopsies, in which case, biomarker testing guidelines in place during the study period [18] recommended testing of these specimens as small biopsies with poorly differentiated areas of squamous histology may actually represent underdiagnosed poorly differentiated adenocarcinoma within an adenosquamous carcinoma. The percentage of ALK rearrangements in squamous histology as observed in our cohort is similar to a recent report in a smaller cohort [19].

We also examined the prevalence of ALK rearrangements as it related to patients deemed eligible for testing as per the NCCN guidelines in place during the study period [18]. The ALK positivity rate was greatest (9.3%) for non-smoking patients with non-squamous histology. Patients with squamous histology who were eligible as per guidelines (generally either nonsmokers or small biopsies) still had a 3.3% positivity rate, underscoring the importance, due to lung cancer heterogeneity, of not overlooking testing in patients with a diagnosis of squamous lung cancer on biopsy. Also, while a low rate of ALK positivity (1.7%) in patients with adenocarcinoma and a smoking history was observed, when coupled with the number of patients with both of these characteristics, this translates to a potentially large number of patients who would be a candidate for TKIs if appropriately identified through testing. In fact, these patients’ rate of ALK positivity (1.7%) is similar to the number of patients expected to have RET or ROS1 fusions [14, 16, 20–23]. The lowest number of ALK rearrangments (0.7%) were observed in patients with a history of smoking and squamous cell carcinoma. At the time of testing, these patients would have been ineligible for testing per NCCN guidelines in place. It is unknown as to why these patient specimens were sent for additional testing but it is likely that these were patients in which multigene panel testing was performed regardless of histology. Given that testing rates in patients with a history of smoking and squamous cell histology have increased in recent years in the US [24, 25], it is not surprising to find a low percentage of patients with ALK rearrangements. Because of lung cancer heterogeneity, in patients with squamous cell carcinoma diagnosed in small biopsies, NGS for actionable targets should be undertaken. Elderly patients and a substantial smoking history could potentially avoid testing but clinicians should use best judgment and if there are any questions about whether to test or not they should err on the side of testing. Younger patients should also be tested regardless of histology and smoking history. The presence of actionable co-mutations or rearrangements on diagnosis is relatively rare and was confirmed in this dataset [26–28].

These findings strongly reinforce the necessity of testing aNSCLC patients for ALK positivity regardless of the previously-depended upon clinical criteria of smoking history and tumor histology. Continuing to use these clinical criteria will deny some aNSCLC patients the opportunity to benefit from therapeutic improvements provided by ALK targeted therapy. NCCN guidelines were updated earlier this year with a recommendation to consider mutational testing in squamous histology without regard to smoking history [29]. This is a good step towards identifying all patients with a possible actionable mutation.

As tempting as it might be to start patients on immunotherapy in the presence of PD-L1 positivity, the number of patients we found who were both ALK positive and PD-L1 positive (3.0%) underscores the need to test for actionable alterations prior to initiation of immune checkpoint inhibitors. ALK positive patients benefit more from TKIs and one cannot simply assume that positive PD-L1 expression rules out the presence of a driver mutation. This underscores the vital need to continue to work nationally to shorten the molecular testing turnaround time for aNSCLC patients, both in academic and community practices.

Our study has the usual limitations of retrospective analyses of EHR databases, such as the potential for coding errors in oncology practices and missing data if procedures or treatments occurred outside the specific oncology practice. It is not possible to discern reasons for failure to test; certainly, some patients might not have been tested in the past due to having a very large burden of disease or impending hospice admission. Likewise, it was not possible to determine why patients with squamous histology and a history or smoking were tested. However, given the self-selection that often takes place with patients seeking care in major academic centers in big cities, having a broader population from primarily community centers from throughout the country provides a better snapshot of the actual incidence of ALK rearrangements in patients with aNSCLC. We are also unable to determine the sequence of testing for the various biomarkers.

In summary, this retrospective review of nearly 20,000 patients with aNSCLC and tested for ALK in the United States confirms that ALK rearrangements are found more commonly in younger nonsmokers and patients of Asian descent. We did not observe a significant difference in prevalence between males and females. However, a substantial minority of cases are found across all age groups, in patients with a history of smoking as well as patients with squamous histology and no history of smoking. Our findings strongly reinforce the need for earlier broad gene panel testing in all patients with advanced lung non-squamous aNSCLC regardless of clinical features. This approach has the potential to identify the maximum number of patients who could benefit from correct identification of actionable driver alterations, ultimately providing these patients with the maximum benefit of molecular therapeutic regimes.

## MATERIALS AND METHODS

### Study design and data source

This cross-sectional observational study utilized Flatiron Health’s nationwide de-identified database derived from electronic health record (EHR) data originating from approximately 280 US cancer clinics (~800 sites of care). The Flatiron Health database is a longitudinal database, comprising de-identified patient-level structured and unstructured data, curated via technology-enabled abstraction [30, 31]. Institutional Review Board approval of the study protocol was obtained prior to study conduct, and included a waiver of informed consent.

### Study population

Patients with advanced non-small cell lung cancer (stage IIIB, IIIC, or IV by American Joint Committee on Cancer 7th edition) [32] diagnosed from January 2015 to May 2019 were included. Eligible patients were also required to be ≥ 18 years old at aNSCLC diagnosis, have documented ALK testing (irrespective of test methodology) and known smoking status. ALK testing information was obtained from biomarker reports, pathology reports/addendums or physician notes.

### Definitions

ALK translocation testing methodology was retrieved from EHR documentation. Various methods for testing were utilized including fluorescent *in situ* hybridization (FISH), next generation sequencing (NGS), immunohistochemistry (IHC) or other methods such as polymerase chain reaction (PCR) and sequencing other than NGS.

For patients with only one documented ALK result, results were categorized as positive (rearrangement/fusion present), negative (rearrangement/fusion not present) or other (unknown result/indeterminate test). If more than one ALK test result was documented, the preference was to accept a positive result over a negative or unknown test result. This sequence was utilized as it would mimic what is observed in practice, as there is not 100% concordance between different methods of testing [33–36].

Per NCCN guidelines that were in place during this study period, [18] a patient was eligible for ALK testing if they had an adenocarcinoma, large cell or NSCLC not otherwise specified (NOS); patients with squamous cell carcinoma and no history of smoking were also eligible. Patients with squamous histology and smoking history were ineligible for ALK testing under NCCN guidelines during the study time period. Although NCCN guidelines recommended ALK testing for patients with small biopsy specimens or mixed squamous histology, we did not include this in our definition of NCCN eligibility criteria as this data was not well-captured in the database.

The status of other driver mutations or rearrangements (EGFR, KRAS, BRAF or ROS1) as well as PD-L1 status was retrieved from the EHR as reported.

### Statistical analysis

Descriptive statistics were used to summarize patient characteristics. Continuous variables were summarized with mean and standard deviation (SD). Frequency counts and the percentage of patients within each category were reported for categorical variables. To test the differences in ALK rearrangement rates by categorical variables, χ^2^ test was used. All analyses were undertaken using R statistical package version 3.5.3 (The R Foundation, Vienna, Austria). Prevalence of ALK rearrangement was assessed overall and then stratified by patient characteristics such as age, gender, race, smoking status and histology.

## Abbreviations

aNSCLC: advanced non-small cell lung carcinoma
ALK: anaplastic lymphoma kinase
NSCLC NOS: non-small cell lung carcinoma not otherwise specified
TKI: tryosine kinase inhibitor
